# An evaluation of nutrition, culinary, and production interventions using African indigenous vegetables on nutrition security among smallholder farmers in Western Kenya

**DOI:** 10.3389/fnut.2023.1154423

**Published:** 2023-05-15

**Authors:** Emily V. Merchant, Martins Odendo, Norah Maiyo, Ramu Govindasamy, Xenia K. Morin, James E. Simon, Daniel J. Hoffman

**Affiliations:** ^1^New Use Agriculture and Natural Plant Products Program, Department of Plant Biology, Rutgers University, New Brunswick, NJ, United States; ^2^Center for Agricultural Food Ecosystems, The New Jersey Institute for Food, Nutrition, and Health, Rutgers University, New Brunswick, NJ, United States; ^3^Kenya Agricultural and Livestock Research Organization, Kakamega, Kenya; ^4^Academic Model Providing Access to HealthCare (AMPATH), Eldoret, Kenya; ^5^Department of Agricultural, Food and Resource Economics, Rutgers University, New Brunswick, NJ, United States; ^6^Department of Nutritional Sciences, Program in International Nutrition, New Jersey Institute for Food, Nutrition, and Health, Center for Childhood Nutrition Education and Research, Rutgers University, New Brunswick, NJ, United States

**Keywords:** agriculture, behavior change, cooking skills, healthy diets, malnutrition, micronutrients, traditional vegetables, orphan crops

## Abstract

**Introduction:**

Nutrition security continues to worsen in sub-Saharan Africa. Current research is limited on how seasonality may influence the impact of nutrition, culinary, and production interventions on food security, diet quality, and consumption of African Indigenous Vegetables (AIV); a culturally accepted source of micro-and-macronutrients that are easily produced due to their adaptation to the local environment. The objective of this study was to evaluate the programmatic impact of AIV interventions on nutrition security among smallholder farmers.

**Methods:**

In a randomized control trial, five target counties in Western Kenya were randomly assigned to one of four treatments: (1) control; (2) production intervention (PI); (3) nutrition and culinary intervention (NCI); and (4) NCI and PI (NCI/PI). After the counties were randomly assigned to a treatment, 503 smallholder farmers (18–65 years) were selected from participatory farmer groups. The PI consisted of five agricultural production modules delivered between 2016 and 2019. The NCI was delivered twice: (1) household nutrition education (2017) and (2) community culinary training (2019). The NCI/PI included communities receiving both interventions at these time periods. Baseline and endline surveys were administered to all participants once in October 2016 (harvest season) and to all available participants (*n* = 250) once in June to July 2019 (dry season), respectively. The impact evaluation was analyzed by Household Hunger Scale (HHS), Women’s Dietary Diversity Score (WDDS), AIV consumption frequency, and AIV market availability. Statistical tests included descriptive statistics (means and frequencies), paired *t*-test, McNemar’s test, Wilcoxon Signed-Rank test, ANOVA test with Tukey *post hoc*, and χ^2^ test. Open-ended questions were aggregated, and responses were selected based on relevancy and thoroughness of the response to provide context to the quantitative data. A value of *p* < 0.05 was used to denote statistical significance.

**Results:**

There was an overall decrease in WDDS, HHS, and consumption frequency between baseline and endline attributed to seasonal differences. Despite this, post-intervention, households that received NCI/PI had a higher WDDS relative to the control: WDDS 5.1 ± 1.8 vs. 4.2 ± 1.5, *p* = 0.035. In addition, between baseline and endline, there was an overall increase in the percentage of respondents that reported an adequate supply of key AIVs, particularly for households that received PI. Furthermore, seasonal effects caused a reported shift in the primary location for purchasing AIVs from the village to the town market. There was no reported difference in HHS. While “diet awareness” significantly influenced diet quality among the NCI treatment group, “production” was reported to have the greatest influence on diet quality among all intervention groups.

**Discussion:**

The findings revealed that coupled nutrition, culinary, and production interventions could create a protective effect against seasonal fluctuations in the availability and affordability of AIV as evidenced by a higher WDDs.

**Conclusion and Recommendations:**

These findings suggest that future programming and policy should focus on promoting the availability, accessibility, acceptability, and affordability of improved agronomic practices and germplasm for both smallholder farmers with particular emphasis on AIV varieties that contain high levels of micro-and macronutrients, improved agronomic characteristics (e.g., delayed flowering, multiple harvests, higher yields, and disease resistance), and are aligned with the communities’ cultural preferences. In addition, agricultural training and extension services should incorporate nutrition and culinary interventions that emphasize the importance of farmers prioritizing harvests for their household consumption.

## 1. Introduction

Food security continues to worsen in sub-Saharan Africa (SSA). The number of undernourished or hungry people in Africa, defined by FAO as those who consume an insufficient number of calories over the course of a year, increased by 46 million between 2019 and 2021 (1). In addition, inadequate intake of micronutrients, such as iron and zinc, are widespread in SSA (2), with half of all anemia cases resulting from dietary iron deficiency (3). The prevalence of undernutrition, which had remained stagnant for many years, worsened due to the COVID-19 pandemic. More specifically, in Kenya, the prevalence of moderate or severe food insecurity in the total population increased from 53% in 2014–2016 to 68.5% in 2018–2020 (1). Poor diets and subsequent inadequate caloric and nutrient intake increases the risk of infectious diseases and micronutrient deficiencies, especially vitamins A and iron, which pose major impediments to social and economic development (1). To improve these diet-related outcomes, it is necessary to design food systems that improve dietary quality and reduce the prevalence of nutrition insecurity, by increasing total energy (calories from macronutrients) and micronutrient intake. Moreover, food system shocks, such as the COVID-19 pandemic are expected to worsen under climate change. Recognizing this major challenge, USAID developed a multi-sectoral nutrition strategy for 2014–2025 to decrease malnutrition, improve nutrition and increase economic productivity (4).

African Indigenous (traditional) Vegetables (AIVs) have unmet potential to contribute to economic and human health in SSA. AIVs are vegetables that either originated or have a long history of cultivation and domestication in Africa and are locally important for economic and human nutrition but have yet to gain regional and global recognition as a major commodity such as carrots or corn (5, 6). AIVs such as African nightshade (*Solanum scabrum*), amaranth (*Amaranthus* spp.), cowpea leaves (*Vigna unguiculata*), and spider plant (*Cleome gynandra*) are culturally accepted through custom, habit, or tradition (7–12) and nutritionally dense (13, 14). In addition, these plants are adapted to the local environmental conditions and often exhibit tolerance to extreme temperatures and precipitation allowing them to be sustainably produced with little to no inputs (8, 15, 16). The combination of these attributes positions AIVs as a rich micro-and macronutrient, climate-resilient food source with unmet economic potential (11). Despite this, limited seasonal availability, poor market access, and high prices impede local production and regular household consumption (8, 9, 17). These difficulties can be overcome by focusing agricultural interventions, programming, and policy on promoting the availability, accessibility, acceptability, and affordability of improved agronomic practices and AIV germplasm, prioritizing high levels of micro-and macronutrients and improved agronomic characteristics (e.g., delayed flowering, multiple harvests, higher yields, and disease resistance) for smallholder farmers. In addition, agricultural training and extension services should incorporate nutrition and culinary interventions that emphasize the importance of farmers prioritizing harvests for their household consumption to ensure nutrition security.

Nutrition security, defined as having consistent access, availability, and affordability of foods and beverages that promote well-being and prevent (and if needed, treat) disease, is required to improve diet-related health outcomes (18). Education on healthy nutrition, good eating habits, food preparation, and safe handling are among effective strategies for overcoming malnutrition and chronic diet-related diseases, such as obesity, diabetes, hypertension, and cardiovascular diseases (19–22). In addition, agricultural interventions that promote the production of nutritionally dense foods such as AIVs can improve access, and availability, and increase household income either through generating income from the sale of produce or saving income from food expenditures (11, 23–25). Current research and literature, however, is limited on how seasonality may influence the impact of nutrition, culinary, and production interventions on nutrition security, diet quality, and consumption of AIVs among smallholder farmers in Western Kenya.

Through a randomized control trial (RCT), this study aimed to evaluate the programmatic impact of a nutrition, culinary, and production interventions on nutrition security during the dry season, in comparison to the harvest season, among smallholder farmers in Western Kenya. We hypothesized that overall diet quality and consumption of AIVs will decrease during the dry season; however, we also hypothesized that the treatment groups would have improved dietary outcomes relative to the control.

## 2. Methods

### 2.1. Overview of study setting

This study was part of a larger research initiative to examine the production and consumption of AIVs in Kenya supported by the USAID Feed the Future Innovation Laboratory for Horticulture (9–11, 26). This study engaged five counties in Western Kenya: Kisumu, Nandi, Busia, Bungoma and Trans Nzoia Counties. Kisumu and Nandi County were treated as one unit due to their proximity to Kisumu City (large market access for AIVs). Agriculture is the main economic activity in the study counties (27, 28). The staple food crop, maize, is often consumed as stiff porridge (*ugali*) alongside cooked leaves of AIVs (29). Moreover, the intervention communities had prior exposure to African Indigenous Vegetables (AIVs) innovation programs and training through USAID-funded Horticulture Innovation Programs (12).

### 2.2. Intervention design

**Figure 1:**
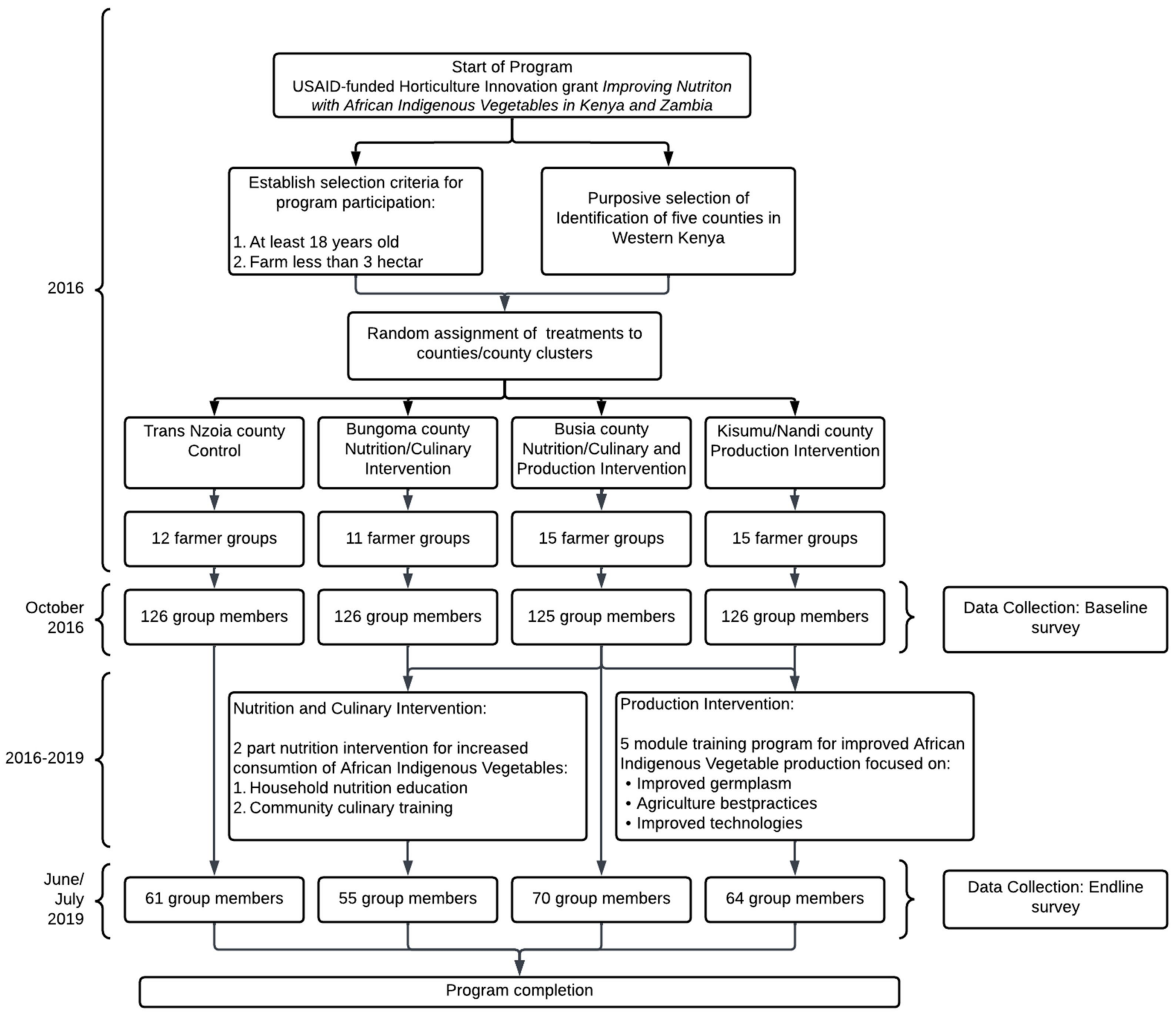
Participant flow diagram.

The study objective was to evaluate the impact of a production, nutrition, and culinary intervention on nutrition security among smallholder farmers in Western Kenya. To that end, the four treatment areas were randomly assigned to one of four interventions summarized below. Figure 1 provides an overview of the program interventions and participant selection.

1. *Control*: No additional intervention or agricultural training.
2. *Production Intervention* (*PI*): Communities received the production intervention, which addressed key bottlenecks in production and distribution, including cultural practices, management, and technologies, improved seed, integrated pest management, irrigation, and drought tolerance management.
3. *Nutrition and Culinary Intervention* (*NCI*): Communities received a two-part nutrition and culinary intervention: (1) promotion of AIV consumption in the households through nutrition education; and (2) community level culinary training. Topics included recommended daily-intake guidelines, recipe and meal-preparation, data on nutrition for each AIV, bodily processes supported, and symptoms of malnutrition alleviated.
4. *PI and NCI:* Communities received both the production and nutrition and culinary interventions.

The five production intervention modules were hosted at demonstration farms central to the intervention communities between October 2016 and May 2019. The modules were delivered by project partners at the Academic Model Providing Access to Healthcare (AMPATH) in Eldoret, Kenya. Each module was delivered within a half to full day session at the demonstration farm. The modules were designed using Good Agricultural Practices evidenced-based best practices developed by the Economic Empowerment and Agricultural division of AMPATH. After each production module was delivered, the trainers would return to the farming community to provide additional support.

The two-part nutrition and culinary intervention were delivered by study members from Kenya Agricultural and Livestock Research Organization (KALRO) and AMPATH as well as locally trained community health workers. Before the nutrition and culinary intervention was delivered, each component was piloted with neighboring communities. July 2017, the first nutrition and culinary intervention was administered at the household level with one or both heads of household. The intervention used a nutrition and culinary pamphlet that was designed from insights gained during a series of focus group discussions with households in the target communities (9). In May 2019, the second nutrition and culinary intervention was delivered at the community level. The intervention households were grouped into clusters of 20, based on location and invited to participate in a community cooking day. As requested by the study community (9), the intervention focused on improved cooking methods and further developing cooking skills such as cooking dried AIV leaves, reduced cooking time, and mixing ingredients.

### 2.3. Sampling and randomization

**Figure 2:**
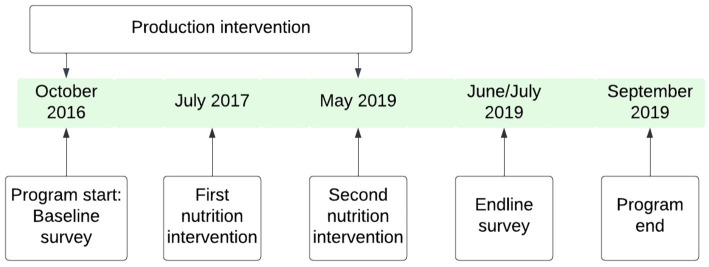
Program timeline.

Before the start of the project, five counties were purposively selected for implementation of the interventions based on prior work with the community groups by project team members. Each of the study county or cluster of counties was randomly assigned to one of the four treatments. We obtained lists of 53 farmer groups and lists of group members from intervention implementation partner (AMPATH; Table 1). All members of the participating farmer groups were allowed to participate in the interventions; however, only a subset participated in the baseline and endline surveys. The lists of group members formed the sampling frame from which, 5–12 individual members were randomly sampled proportionate to the group sizes for inclusion in this study ensuring that the study was representative among smallholder farmers in Western Kenya. The group members were assessed for inclusion eligibility based on the following criteria: the group member was from a household had a primary farmer, male or female aged 18–65 years, and owned a small farm or garden (defined as <1 ha). In addition, the household had to have a woman over 18 years to participate in the Women’s Dietary Diversity questionnaire. Exclusion criteria include any households that were horticultural farmers or commercial farmers cultivating/managing land more than 3 ha regardless of whether they had a small kitchen garden. Figure 2 provides an overview of the timeline of the program interventions.

**Table 1:** Assignment and number of households to the four intervention treatments in Western Kenya.

			Number of surveyed households	
Treatment	County	Sub-county(s)	Baseline (2016)	Endline (2019)
Control	Trans Nzoia	Kiminini	126	61
Production intervention	Nandi	Nandi South	41	20
	Kisumu	Kisumu West	85	44
Nutrition and culinary intervention	Bungoma	Sirisia, Webuye East, and Webuye West	126	56
Production and Nutrition/culinary intervention	Busia	Matayos and Teso South	125	69
Total (*n*)			503	250
Attrition *n* (%)				253 (50)

A total of 503 households, across counties, were randomly selected using a random sampling procedure from farmer group members’ lists to participate in the baseline survey before roll-out of the project interventions. At endline, 250 households, across counties (Figure 2), who were interviewed during the baseline survey also participated in the endline survey. The attrition between baseline and endline surveys was mainly attributed to respondents not being found at home at the time of the survey for several reasons despite at least one repeat visit or appointment. The main reasons included involvement of potential respondents in off-farm businesses, attending social functions such funerals and community meetings, separation or divorces in the households, migration, fall-out of members from their groups and death. A high attrition was anticipated due to instability in the rural area and potentially difficulty access communities due to extreme weather (30, 31). No incentives or gifts were given to any of the treatment or non-treatment groups for participation in the survey or research. However, after the intervention, a small quantity of AIV seeds were given to all households who had participated in the surveys.

### 2.4. Survey instrument

The baseline and endline structured questionnaire were developed by Rutgers University, United States in collaboration with KALRO in English and translated into the local languages. The questionnaires contained nine sections: (1) identifying respondents and the study area; (2) household demographics; (3) household living conditions; (4) household general food consumption frequencies; (5) consumption and utilization of AIVs; (6) a modified FANTA III Household Hunger Scale (HHS) (32); (7) consumer attitudes and preferences; (8) FANTA III Women’s Dietary Diversity Score (WDDS) (33); and (9) women’s role in decision making. The endline survey contained one additional section: participant feedback. This section ascertained data relative respondent’s perception of their dietary changes and the reasons for these changes post-intervention, with the later questions being open-ended. A copy of the endline survey can be found in Supplemental material Appendix A.

Due to time constraints, for HHS, this study used a modified FANTA III. The survey focused on three questions [(1) no food of any kind, (2) go to sleep hungry, and (3) go a whole day or night without food] and their subsequent frequency questions. HHS was then calculated for each respondent by summing the points relative to each response. Using the following scores (e.g., 1 for yes; 1 for “sometimes,” and 2 for “often”) each question had a maximum of three points for a total HHS of 0 (low household hunger) to 9 (high household hunger).

For WDDS and AIV consumption, at an individual level, the eldest female in the household was asked whether they consumed numerous food groups, from an extensive list of food groups and specific foods (e.g., different AIVs) on a regularly basis within a 24-h period. The food groups were then aggregated into the following nine categories used to calculate WDDS: starchy staples; dark green leafy vegetables; other fruits and vegetables; organ meat; meat and fish; eggs; legumes, nuts, and seeds; and milk and milk products. WDDS was calculated by summing the total number of food groups consumed from 0 (low dietary diversity) to 9 (high dietary diversity) (33). In addition, AIV consumption was calculated by summing the total number of AIVs consumed from 0 (low AIV consumption diversity) to 11 (high AIV consumption diversity).

Reported food frequencies were recoded as follows: never = 0, sometimes = 1, once a month = 0.25, and everyday = 7. The questionnaire ascertained data relative to food consumption frequency for 28 different foods with a possible total of seven points per food for a total of 196 points. Post-intervention, the following food frequency tertials were calculated to group the respondents: low = 0–29.25; middle = 29.26–36.25; and high = 36.36–196.

### 2.5. Data collection

Each participant included in the study completed a total of two surveys: (1) baseline and (2) endline. The baseline and endline survey were conducted in October 2016 (wet season) and in June and July 2019 (dry season), respectively. Data were collected through face-to-face interviews by a team of locally recruited and trained enumerators. Prior to the interview, all potential respondents were made aware of the purpose of the survey as well as the overall objective of the study. The questionnaire was administered in *Kiswahili, Luhya,* and *Luo* dialects. The implementation of field data collection was overseen by lead KALRO collaborating agricultural economist, who monitored completeness and consistency of the responses to ensure that all sections of the questionnaires had been answered appropriately.

The data were validated by the lead agricultural economist and uploaded to the server and downloaded in Excel Software for ease of management and analysis on a daily basis. The data were matched with the baseline data using the same household code. Data were cleaned and exploratory checking conducted to identify key anomalies and outliers. The data were anonymized using codes of respondents, which were only known to lead economist. For confidentiality of respondents, data with no identifiers were shared with Rutgers University collaborators for analysis.

Data were collected by 14 enumerators. The enumerators were selected based on educational background, prior experience, knowledge of the local language, and familiarity with the areas where data collection was to occur. A total of 14 enumerators were trained at a central venue where they were taken through the content of the questionnaire. Additional training topics included understanding the objectives of research, understanding questionnaire content, framing of questions, and field implementations and procedures. Additionally, the enumerators and those handling the data were each trained and received *Collaborative Institutional Training Initiative* (*CITI* Program) certification prior to survey initiation.

Before embarking on the final data collection, the enumerators and the supervisors pretested the questionnaire with households near the training venue (Busia Agricultural Training Center) to ensure that the enumerators fully understood the content of the questionnaire, the order of the questions, and the skip patterns. At endline, the questionnaire was programmed in Open Data Kit (ODK) platform and loaded in the mobile phones. Enumerators received additional training on the use of mobile phones and applications for data collection.

### 2.6. Data analysis

Descriptive statistics (means and frequencies) were used to summarize respondent data. A paired *t*-test and a McNemar’s test was used to examine the statistical differences, either in continuous or in categorical data respectively, in means between timepoints within the same sample population (e.g., baseline and endline analysis per treatment group). A Wilcoxon Signed-Rank test was used to examine the statistical differences in means for WDDS due to a lack of normal distribution.

Using only the post-intervention data, an ANOVA test was used to examine the differences in means and treatment groups. A Tukey *post hoc* ANOVA test was used to determine the statistical differences between groups. In addition, post-intervention, a χ^2^ test was used to calculate the statistical differences between categorical data and treatment groups. Open-ended questions were aggregated, and responses were selected based on relevancy and thoroughness of the response to provide context to the quantitative data.

Pairwise deletion was used to handle cases of missing data. Quantitative analyses were conducted using SPSS (IBM SPSS Statistics v 26; Armonk, New York, United States) and a *p* value of <0.05 was used to denote statistical significance.

### 2.7. Ethical considerations

Ethical approval in the United States was provided by the Institution Review Board at Rutgers University, the State University of New Jersey. Ethical approval in Kenya was provided by the Institutional Research and Ethics Committee at the Academic Model for Providing Access to Healthcare (AMPATH) in Kenya. Before beginning the interview, the enumerators introduced themselves and assured participants that the survey was voluntary and that there would be no disclosure of individual information. All study participants provided informed oral consent to participate in the study and for use of the data in publications. The use of oral consent was approved by the ethical review boards due to minimal associated risk and low literacy rates among the study population.

## 3. Results

### 3.1. Demographic

Due to program attrition, a total of 250 households were included for analysis. Table 2 provides an overview of the demographic characteristics of the study population. Most households had both male and female heads of households and male heads of households were an average 51.1 years old. In addition, the majority of heads of households were married and both male and female heads of household had primary education. Households had an average size of seven members, had three sources of income, and consumed three meals per day; however, the control group reported consuming significantly more meals compared to PI and NCI/PI intervention groups at baseline (*p* = 0.001 and *p* = 0.005 respectively).

**Table 2:** The 2016 demographic information of the surveyed households in Western Kenya.

	Control	NCI	PI	PI and NCI	All households	*p* value^1^
*N*	61	55	64	70	250	
County	Trans Nzoia	Bungoma	Kisumu and Nandi	Busia		
Heads of household (HH) (%)						
*Only male*	3.3	12.7	18.8	14.3	12.4	0.063
*Only female*	24.6	18.2	32.8	17.1	23.2	0.133
*Both male and female*	72.1	69.1	46.9	68.6	64.0	0.011^*^
Male HH Age (mean ± SD)	55.5 ± 11.7	52.8 ± 13.0	48.2 ± 13.4	48.7 ± 15.5	51.1 ± 13.8	0.034^*^
HH marital status (%)						0.149
*Married*	83.6	81.8	68.8	81.4	78.8	
*Widowed*	13.1	14.5	26.6	11.4	16.4	
*Other*	3.3	0	1.6	4.3	2.4	
*Missing*	0	3.6	3.1	2.9	2.4	
HH education (%)						0.010^*^
*None*	3.3	5.5	4.7	4.3	4.4	
*Primary*	41.0	50.9	54.7	65.7	53.6	
*Secondary*	39.3	43.6	29.7	25.7	34.0	
*University and higher*	14.8	0	7.8	0	6.0	
*Missing*	1.6	0	3.1	2.9	2.0	
HH spouse education (%)						0.004^*^
*None*	0	5.5	4.7	7.1	4.4	
*Primary*	39.3	45.5	53.1	60	50.0	
*Secondary*	41.0	34.5	14.1	18.6	26.4	
*University and higher*	1.6	0	6.3	1.4	2.4	
*Missing*	18.0	14.5	21.9	12.9	16.8	
Household size (mean ± SD)	6.9 ± 2.8	6.5 ± 1.9	6.4 ± 2.8	6.5 ± 2.4	6.6 ± 2.5	0.695
Sources of income (mean ± SD)	2.9 ± 0.8	2.6 ± 0.8	3.0 ± 0.6	2.6 ± 0.8	2.8 ± 0.7	0.010^*^
Number of meals consumed prior 24 h (mean ± SD)	3.4 ± 0.9	3.2 ± 0.7	3.0 ± 0.4	3.0 ± 0.7	3.1 ± 0.7	0.001^*^

NCI, nutrition/culinary intervention; PI, production intervention.

1 Continuous variables (e.g., age, household size) between sample populations were compared using one way ANOVA with Tukey post hoc and categorical variables were compared using chi square.

* *p* < 0.05.

### 3.2. Intervention influence on diet quality

Overall, there was a significant decrease in dietary diversity (*p* < 0.001) and an observed decrease in other outcome variables post-intervention (Table 3). Post-intervention, the NCI/PI intervention group reported a significantly higher WDD score relative to control: 5.1 ± 1.8 vs. 4.2 ± 1.5, *p* = 0.035. However, this group also reported a significantly higher household hunger score post intervention relative to control: 0.9 ± 1.5 vs. 0.3 ± 1.0, *p* = 0.05. The intervention groups that received the nutrition intervention (NCI and NCI/PI) reported the highest proportion of participants whose food frequency score was within the lowest tertial. In addition, the PI intervention group reported the highest proportion of participants whose food frequency score was within the middle tertial.

**Table 3:** Baseline and endline study outcome variables.

	Baseline	Endline	*p* value^1^
Women’s dietary diversity score	7.9 ± 1.5	4.6 ± 1.7	< 0.001^**^
Household hunger score	0.5 ± 1.4	0.6 ± 1.3	0.48
Food frequency score	37.0 ± 18.4	34.8 ± 11.6	0.099

1 *p* value for women’s dietary diversity score calculated using Wilcoxon Signed-Rank test and household hunger score and food frequency score calculated with paired *t*-test.

** *p* < 0.01.

**Figure 3:**
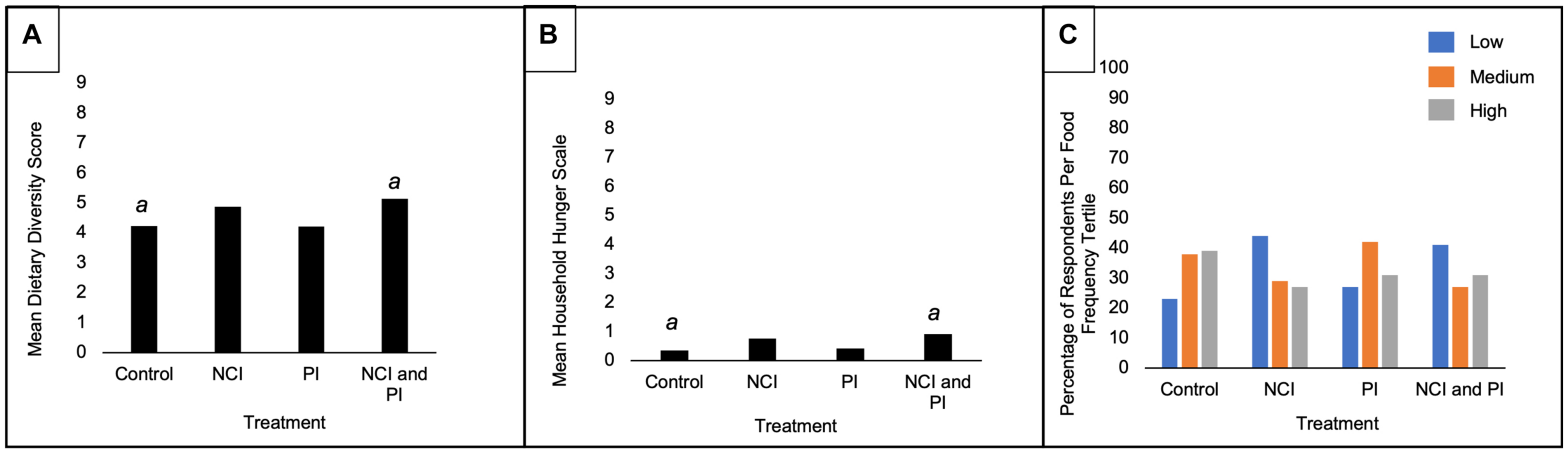
Post-intervention outcome variables by treatment interventions. NCI, nutrition and culinary intervention; PI, production intervention. ^a^Statistically significant difference between treatments at *p* < 0.05 (ANOVA Tukey *post hoc*). **(A)** Mean Women’s Dietary Diversity Score. **(B)** Mean Household Hunger Score. **(C)** Percentage of respondents per food frequency tertial.

**Figure 4:**
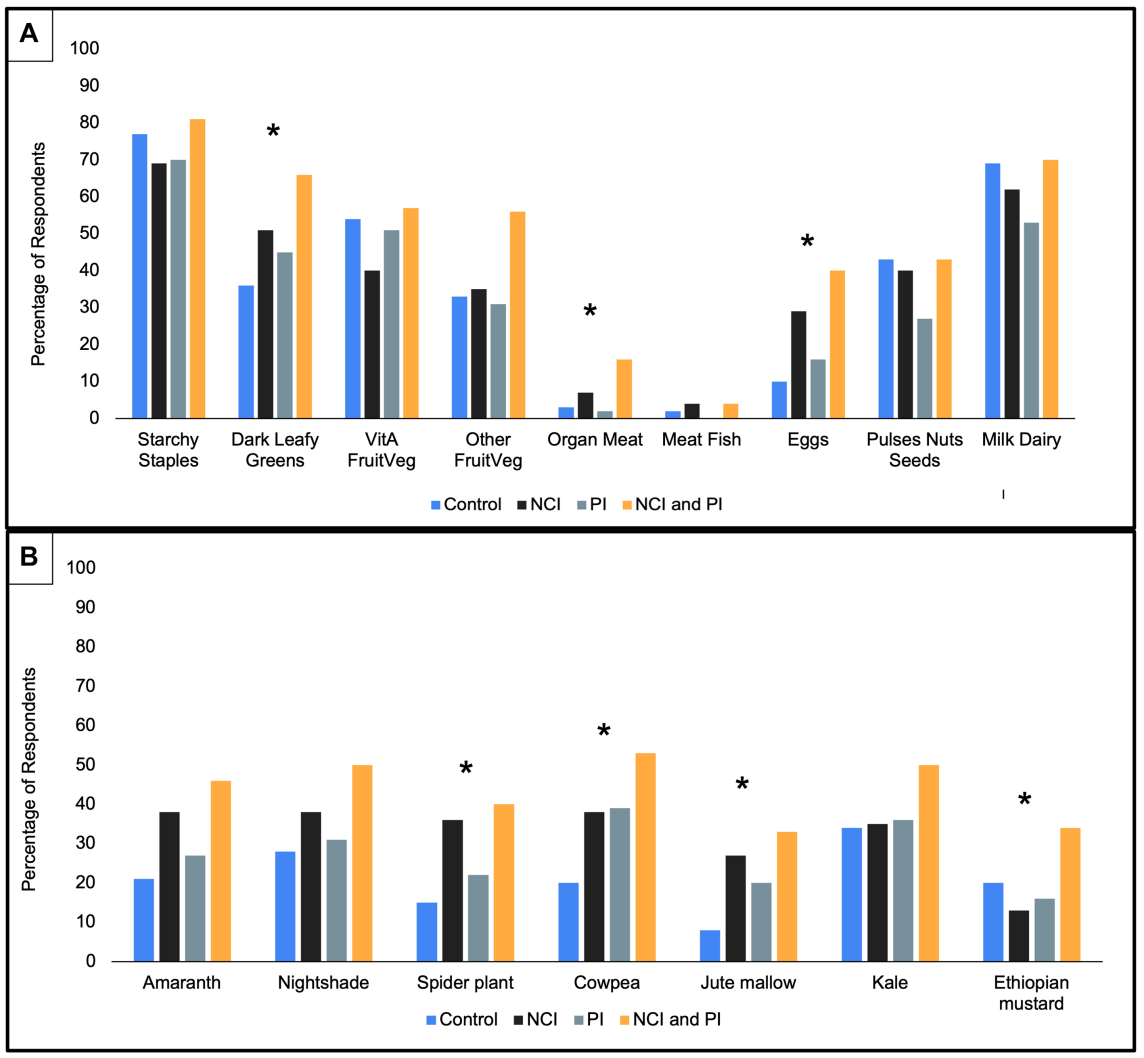
Food consumption by treatment interventions at endline. ^*^Statistically significant difference between treatments at *p* < 0.05 (χ^2^ test). **(A)** Food groups consumed in typical 24-h period. **(B)** African Indigenous Vegetables consumed in typical 24-h period.

At endline, there was a significant difference in consumption patterns between the intervention groups (Figure 3). The NCI and NCI/PI intervention groups, respectively, reported a significantly higher percentage of respondents consuming the following food groups and AIVs within a 24-h period relative to control (Figure 4): dark leafy greens (51 and 66% vs. 36%, *p* = 0.003), organ meat (7 and 16% vs. 3%, *p* = 0.016), eggs (29 and 40% vs. 10%, *p* = 0.001), spider plant (36 and 40% vs. 15%, *p* = 0.004), and jute mallow (27 and 33% vs. 8%, *p* = 0.007). Moreover, all intervention groups reported consuming significantly more cowpea relative to control (*p* = 0.001); however, NCI group reported consuming significantly less Ethiopian mustard relative to the other treatment groups (*p* = 0.05).

### 3.3. Source and supply of food items post-intervention treatment

**Figure 5:**
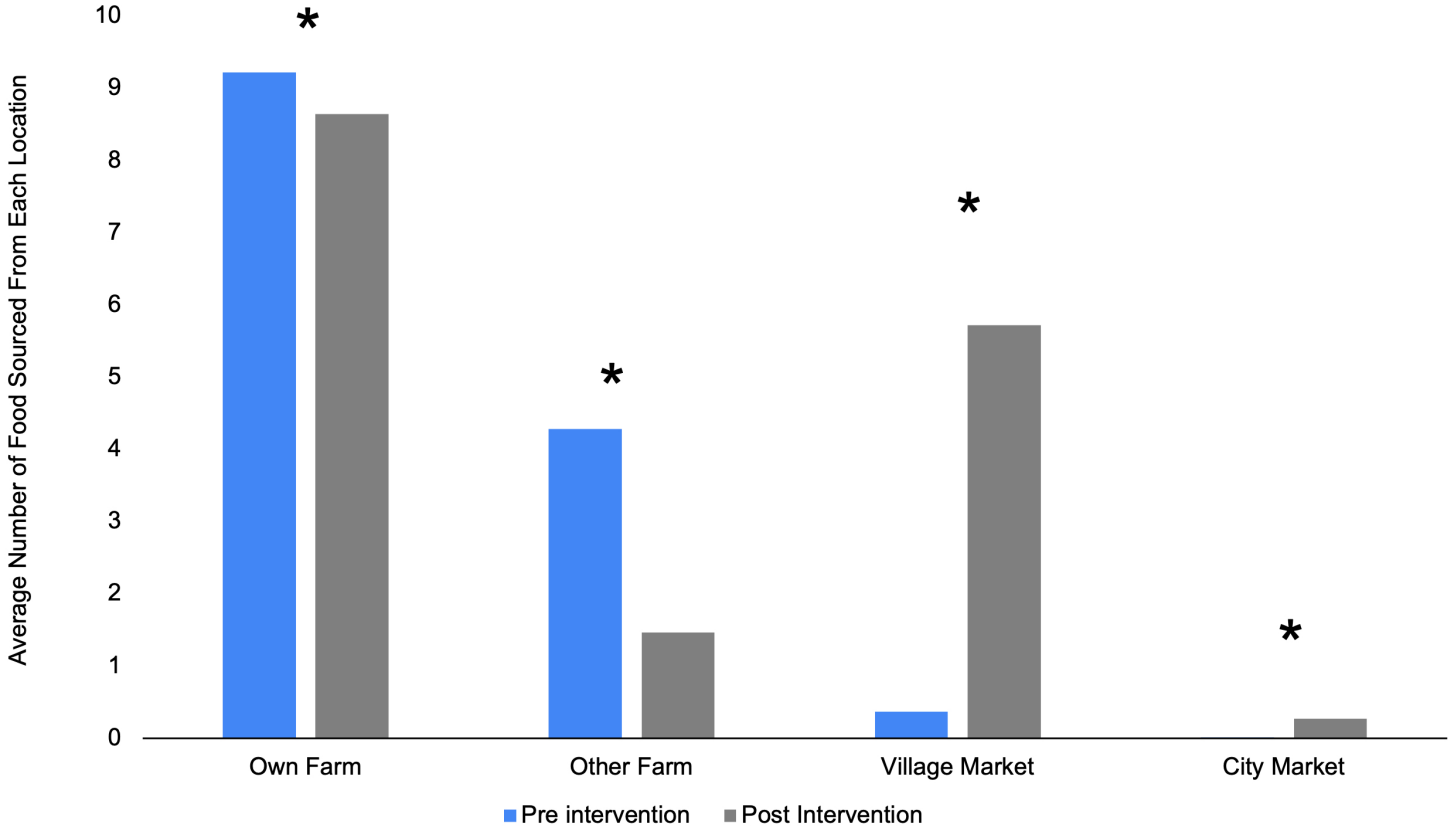
Average number of food items sourced from each location, overall, pre-and post-treatment interventions. ^*^Statistically significant difference between baseline and endline at *p* < 0.05 (paired *t*-test).

Prior to the intervention, respondents reported sourcing the highest number of food items from their own or other farm (Figure 5). Post-intervention, there was a significant shift in locations used to source food items, with a decrease in all reported locations except village market. Post-intervention, respondents reported acquiring over five items, on average, from the village market, compared to less than one at baseline (*p* < 0.001). Moreover, there was a significant increase in individuals reporting sourcing food items from the city market (*p* < 0.001).

**Figure 6:**
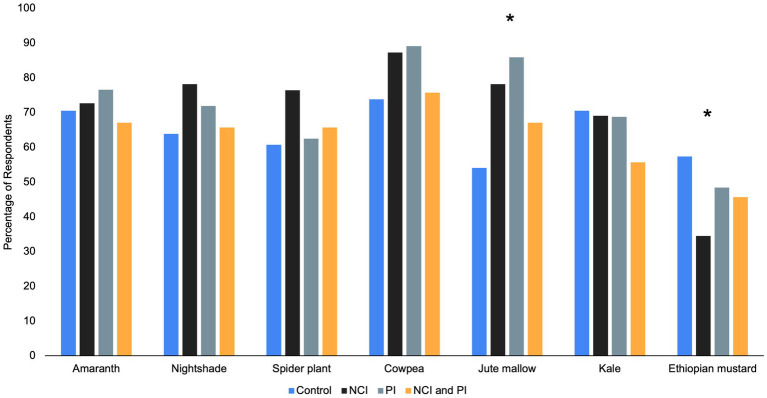
Percentage of respondents who reported an adequate supply of African Indigenous Vegetables post-intervention. ^*^Statistically significant difference between treatments at *p* < 0.05 (χ^2^ test).

**Figure 7:**
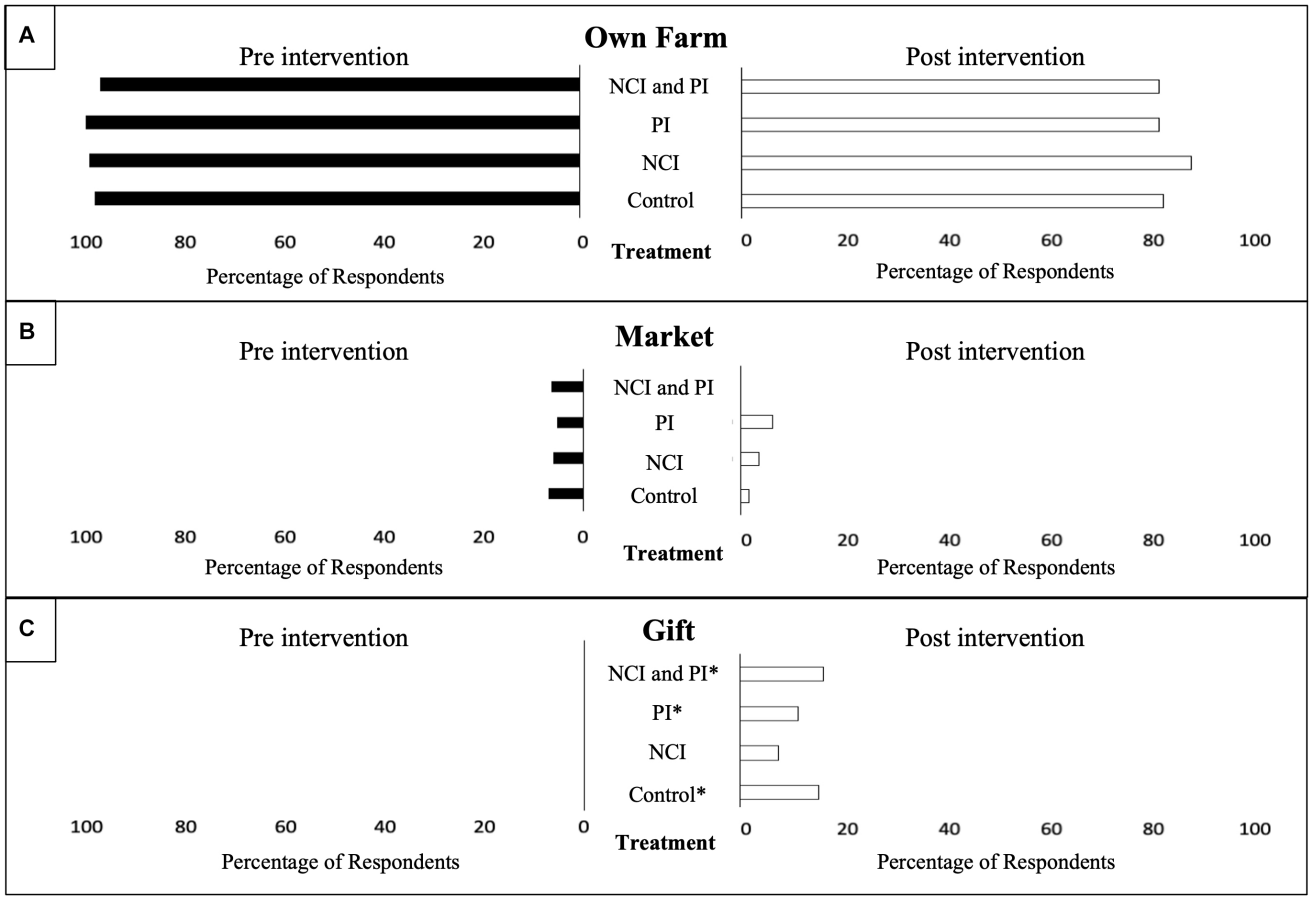
Reported supply of African Indigenous Vegetables (AIVs) by treatment pre-and post-intervention. ^*^Statistically significant difference between baseline and endline at *p* < 0.05 (McNemar’s test). **(A)** Percentage of respondents sourcing AIVs from their own farm. **(B)** Percentage of respondents sourcing AIVs from the market. **(C)** Percentage of respondents sourcing AIVs as a gift.

**Figure 8:**
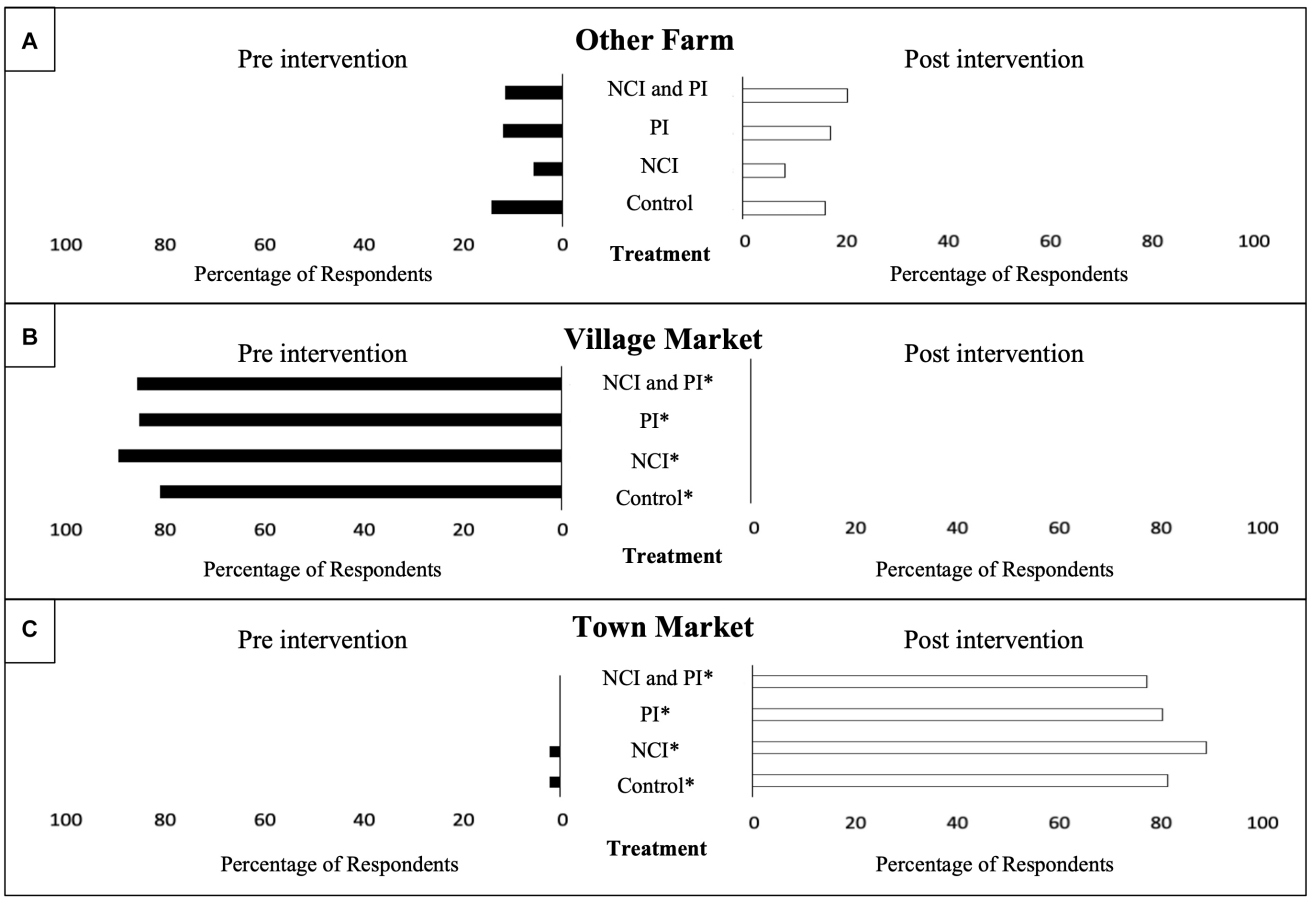
Purchasing place of African Indigenous Vegetables (AIVs) by pre-and post-treatment interventions. ^*^Statistically significant difference between baseline and endline at *p* < 0.05 (McNemar’s test). **(A)** Percentage of respondents purchasing AIVs from other farms. **(B)** Percentage of respondents purchasing AIVs from the village market. **(C)** Percentage of respondents purchasing AIVs from the town market.

Post-intervention there were differences in the percentage of respondents who reported an adequate supply of various AIVs between treatment groups (Figure 6). There was a significant difference in reported supply for jute mallow (*p* = 0.002) and Ethiopian mustard (*p* = 0.001) between treatment groups. The highest percentage of respondents in both the NCI and PI intervention groups reported an adequate supply of jute mallow; however, the NCI group reported an inadequate supply of Ethiopian mustard. When respondents were asked the main source AIVs, respondents in the NCI/PI, PI, and control intervention groups noted a significant increase post-intervention to receiving AIVs as gifts (*p* = 0.004; *p* = 0.016; and *p* = 0.004 respectively; Figure 7). Moreover, when respondents were asked the main location for purchasing AIVs, all treatment groups reported a significant decrease in purchasing AIVs at the village market pre-intervention (*p* < 0.001 all groups) to the town market post-intervention (*p* < 0.001 all groups; Figure 8).

### 3.4. Perception of dietary change post-intervention treatment

**Figure 9:**
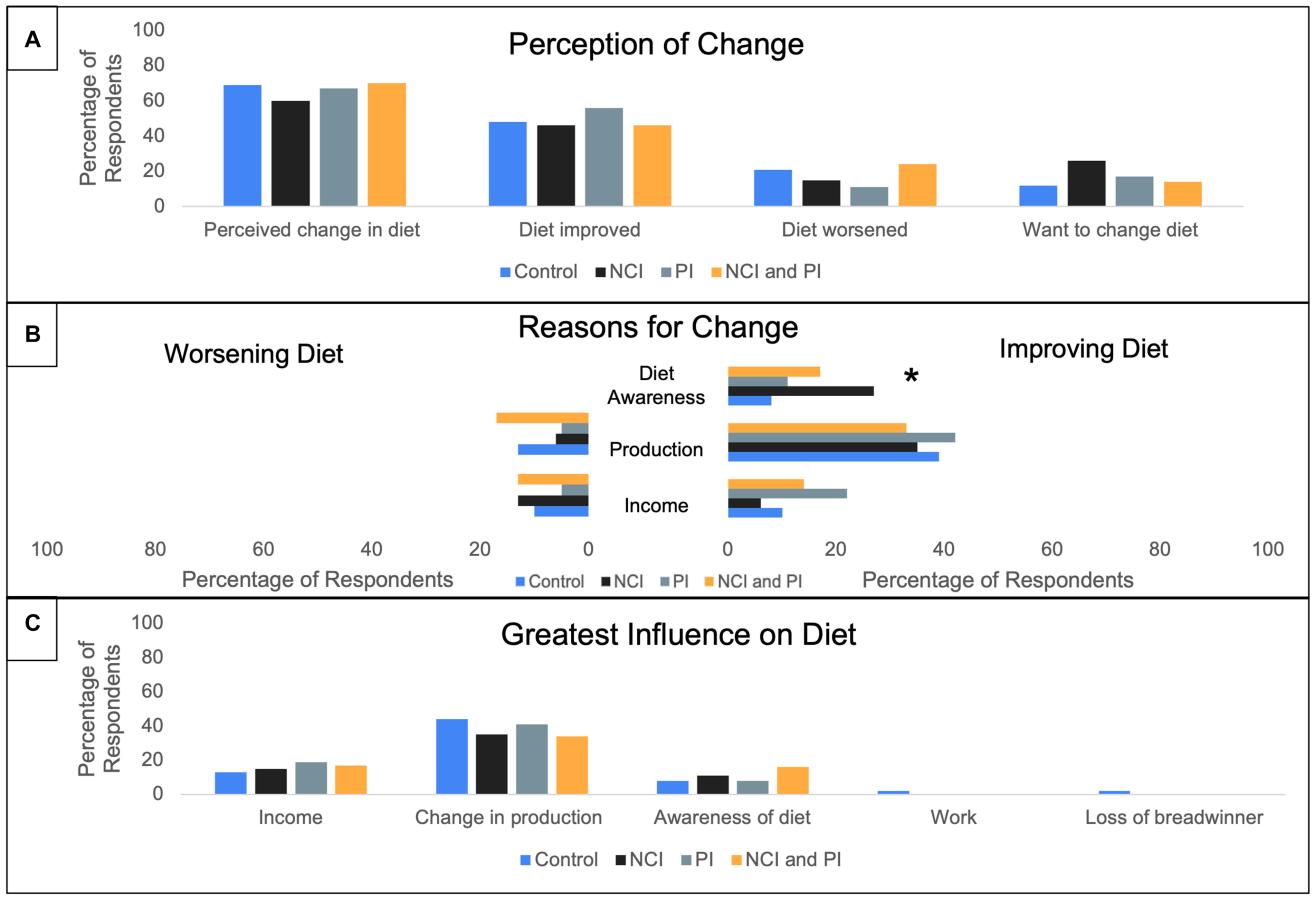
Percentage of respondents perceiving dietary changes post-treatment interventions. Multiple answers recorded. ^*^Statistically significant difference between treatments at *p* < 0.05 (χ^2^ test). **(A)**. Percentage of respondents who reported a perception of dietary change. **(B)** Reported reasons for diet either worsening or improving. **(C)** Reported greatest influences on dietary change.

Respondents reported perceptions on dietary changes post-intervention by treatment (Figure 9). Post-intervention a majority of respondents, across treatment groups, reported a perceived improvement in diet quality. There was significant difference among treatment groups (*p* = 0.011) who reported “diet awareness” as the reason for improving diet with the highest reporting from the NCI treatment group. In addition, “production” and “income” were reported as reasons for improving diet quality. Moreover, regardless of treatment group, respondents noted that “change in production” had the greatest influence on diet. When respondents were asked to expound on the reasons that limited their ability to change their diet, they cited “lack of enough resources,” “increased school fees,” “loss of breadwinner,” “lack of knowledge in preparing the vegetables in a better way,” “knowledge,” and “lack of money.” When respondents were asked to explain reasons for their diet improvement “sufficient rain” and “few pests and diseases in crops” were reported.

## 4. Discussion

This study sought to examine the impact of nutrition, culinary, and production interventions on food security, diet quality, and AIV consumption in Western Kenya. Overall, there was a significant decrease in WDDS, HHS, and consumption frequencies during the dry season. However, the nutrition, culinary, and production interventions posed some protective effect on diet quality. In addition, there was a reported shift in adequate supply and source location post-intervention. Despite a desire to change one’s diet, and nutrition and culinary awareness, production remained the greatest influence on AIV consumption post-intervention.

### 4.1. Diet quality and AIV consumption patterns

Overall, seasonal differences resulted in an overall decrease in WDDS, HHS, and consumption frequency between baseline and endline. This coincides with existing literature where near daily consumption of AIVs was reported during peak seasons. However a decrease in consumption frequency as low as once a week was reported during off-seasons (34). Post-intervention, households that received both the nutrition/culinary and production interventions demonstrated a protective effect as measured by a higher WDDS relative to the control. Self-production, nutritional awareness, and necessary culinary skills gained in the NCI/PI intervention group could offset the reported limited availability and associated increase in market price reported during the dry season (35–37). Furthermore, income generated from selling surplus AIVs in the dry season could be used for household expenditures such as food (24). It is common for producers to favor sales over household consumption (38). This is further evidenced in this study by the significantly higher HHS in the NCI/PI intervention group relative to control. Therefore, it is important that interventions emphasize the importance of farmers prioritizing harvests for their household consumption.

In addition, in the NCI/PI intervention group, the reported high dietary diversity with corresponding high household hunger could be due to using a modified FANTA III survey to evaluate HHS. The HHS questions ascertained data relative to more severe household hunger (e.g., no food of any kind, go to sleep hungry, and go a whole day or night without food). While household members in this study area may have experienced these conditions within a 30-day period, on a typical day within a 24-h period, they may have also consumed a diverse diet leading to a high WDD score. Moreover, had the HHS questions ascertained data relative to low or moderate hunger such as whether household members ate smaller portions or ate food they would rather not eat, the data may have revealed different HHS scores in the different treatment groups.

### 4.2. Source and supply of food items

Post-intervention, respondents reported an overall increase an adequate supply of key AIVs, particularly for households that received the production intervention. AIV production is vulnerable to environmental stressors such as drought. However, this aggregate group of plants, which come from different plant families and thus differ genetically, do tend to be less sensitive to shocks as compared to “Western” introduced crops such as cabbage. This is also evidenced by each being found in the wild and successfully naturalized across many different environments. This positions AIVs as a climate-resilient commodity and stabilizes their availability throughout the year (8, 15, 39). In addition, the complementary agricultural training and continued extension support, which is often unavailable to smallholder farmers (40), could have strengthened their local value chain during the dry season. For example, a study in Vietnam by Sattaka et al. (41) reported that agricultural extension services influenced the production of culturally preferred glutinous rice and helped to ensure local food and cultural security. Moreover, the availability of improved germplasm and seed stock for producers could have improved production and yield (42–45). For example, a study by Ojiewo and colleagues (46) found a significant difference in the yielded dry weight, plant height, and flowering time between different varieties of African nightshade. Furthermore, with such rich germplasm, targeted breeding could screen existing germplasm and select for varieties that can withstand climate shocks such as extreme drought. In addition to influencing on-farm availability, climate variability can limit affordability, and accessibility within the larger value-chain.

Across intervention groups, there was a reported shift from sourcing AIVs from village to town market. Smallholder farmers often supply the local village markets and have historically been unable to access the larger commercial value-chain (47). With seasonal drought impeding on-farm production and subsequently decreasing supply to the local village markets, households would be forced to travel to the larger town market, supplied by commercial farmers, to procure the household foods. A study in Kenya by Gido et al. (48) found that retail outlets followed by farm gate outlets were the most preferred food sources in rural households while green groceries were the least preferred. In addition, proximity to household was a major determinate in retail preferences (48). Post-intervention, increased sourcing of food from the town markets, compared to pre-intervention, underscores the limited availability and affordability of AIVs within the local value-chain.

### 4.3. Perception of dietary change post-intervention treatment

Despite a desire to change one’s diet, and regardless of intervention, respondents perceived that production remained the greatest influence on AIV consumption post-intervention. The nutrition and culinary interventions were designed in response to community needs using focus group discussions. Moreover, the nutrition and culinary intervention group noted that diet awareness significantly improved diet quality; yet even this intervention group reported production as the single greatest influence on diet quality. AIV production can contribute to household food security and sovereignty, and ecological sustainability through household and community autonomy within the food system (38, 49–52). A study in Petén, Guatemala by Marquez and Schwartz (53) reported that diverse and productive home gardens, contributed to household income, nutrition delivery, and strengthened social bonds and networks. In addition, a review study by Garcia et al. (54) revealed that community cooking classes increased confidence in cooking skills and promoted the consumption of fruits and vegetables.

### 4.4. Limitations

This study relied on recall data and therefore, it is possible that in certain cases the data collected may not be accurate as many participants do not keep written records. However, in such cases probing questions were asked to get the most possible accurate data from the respondent. In addition, additional corresponding data collected during a subsequent rainy or dry season for comparison could provide further insights on programmatic impact. Lastly, the use of the full validated FANTA IIII household hunger survey tool could have revealed a more nuanced understanding of diet quality food insecurity in the target communities.

## 5. Conclusion and recommendations

This paper examined the programmatic influence of nutrition, culinary, and production interventions on nutrition security among smallholder farmers Western Kenya. The findings revealed that coupled nutrition, culinary, and production interventions could create a protective effect against seasonal fluctuations in the availability and affordability of AIV as evidenced by a higher Women’s Dietary Diversity Score (WDDs). Furthermore, post-intervention, a higher proportion of respondents reported adequate supply of AIVs; however, when they needed to purchase AIVs there was a shift to the town market potentially due to limited availability, accessibility and or affordability at the village market, the preferred retail outlet pre-intervention.

Regardless of treatment, respondents perceived agricultural production to have the greatest influence on diet quality, despite the nutrition and culinary intervention group report that ‘diet awareness’ had a significant impact on diet quality. Future research directions include evaluating programmatic impact between similar seasons within smallholder farmer groups. Future programming and policy should focus on promoting the availability, accessibility, acceptability, and affordability of improved agronomic practices and germplasm for both smallholder farmers. In addition, agricultural training and extension services should incorporate nutrition and culinary interventions that emphasize the importance of farmers prioritizing harvests for their household consumption. Commercial seed industries, in partnership with regional leaders such as the World Vegetable Center, should prioritize the development improved AIV varieties that contain high levels of micro-and macronutrients, improved agronomic characteristics (e.g., delayed flowering), and are aligned with the communities’ cultural preferences.

## Data availability statement

The raw data supporting the conclusions of this article will be made available by the authors, without undue reservation.

## Ethics statement

The studies involving human participants were reviewed and approved by Institution Review Board at Rutgers University, the State University of New Jersey Institutional Research and Ethics Committee at the Academic Model for Providing Access to Healthcare (AMPATH) in Kenya. Written informed consent for participation was not required for this study in accordance with the national legislation and the institutional requirements.

## Author contributions

JS, DH, RG, and XM contributed to study design and survey development in concert with MO. NM led the interventions and participated in the field survey including data collection. MO led the survey implementation, enumerator training, and data management. EM analyzed the data and wrote the manuscript draft. All authors contributed to the article and approved the submitted version.

## Funding

This research was supported by the Horticulture Innovation Lab with funding from the U.S. Agency for International Development (USAID EPA-A-00-09-00004), as part of the U.S. Government’s global hunger and food security initiative, Feed the Future, for project titled “Improving nutrition with African indigenous vegetables” in eastern Africa that was awarded to Rutgers University. The contents are the responsibility of the Horticulture Innovation Lab and do not necessarily reflect the views of USAID or the United States Government. Additional funding was provided by the New Jersey Agricultural Experiment Station HATCH project 12170 and the New Jersey IFNH Center for Agricultural Food Ecosystems at Rutgers University.

## Conflict of interest

The authors declare that the research was conducted in the absence of any commercial or financial relationships that could be construed as a potential conflict of interest.

## Publisher’s note

All claims expressed in this article are solely those of the authors and do not necessarily represent those of their affiliated organizations, or those of the publisher, the editors and the reviewers. Any product that may be evaluated in this article, or claim that may be made by its manufacturer, is not guaranteed or endorsed by the publisher.
