# Supplementary material for: An evaluation of nutrition, culinary, and production interventions using African indigenous vegetables on nutrition security among smallholder farmers in Western Kenya
Source: Front Nutr. 2023 May 15;10:1154423. doi: 10.3389/fnut.2023.1154423 (PMC10225545; doi:10.3389/fnut.2023.1154423)
Supplement: Supplementary file 1 [file Data_Sheet_1.PDF]

Follow Up Household Consumption Survey the African Indigenous Vegetables in Zambia and Kenya

1. IDENTIFYING RESPONDENTS AND THE STUDY AREA

The following questions are only recorded and not asked

1.1.Name of Enumerator: ____________________

1.2.Name of Supervisor: ____________________

1.3.Date of interview: _________________

1.4.Start time of interview: __________________

1.5.GPS Readings: ________________

1.6.County: 1. Busia 2. Bungoma 3.Kisumu 4.Nandi 5. Trans Nzoia

1.7.Sub-county: 1.Matayos 2. Teso South 3.Sirisia 4.Webuye West 5. Webuye East 6.Kisumu West 7.Nandi South 8. Kiminini

1.8.Ward: ______________________

1.9.Village: ____________________

1.10. Household ID___________________

1.11. Ability to be interviewed alone: ***(Circle one)*** **1**.Alone; **2**. With adult females present **3**. With adult males present **4.** With adults mixed sex present **5**. With children present **6**. With adults mixed sex and children.

1.12. . Outcome of interview: ***(Circle one)*** **1.** Completed **2.** Household member too ill to respond/cognitively impaired **3.** Respondent not at home/temporarily unavailable **4.** Respondent not at home/extended absence **5.** Refused **6.** Could not locate

The following questions are to be asked and recorded

1.13.Name of respondent: _____________________________1.1 Mobile phone No _____________

1.14. Sex of respondent (*Only recorded, not asked!*)(*Circle one*) **1.** Male; **0**.Female

1.15. Age of respondent (in years): ______________

1.16 Are you the head of household? (*Circle one*) **1.** Yes **0.** No

If NO continue to 1.17, if YES skip to 1.21

1.17. Relation of respondent to the household head
(***Circle one)*** 1. Spouse; 2. Son; 3. Daughter; 4. Other (specify) _______

1.18.a Name of household head (HH): _________________________1.18b. Mobile phone No ___________

1.19. Sex of HH head: [**1**] Male [**2**] Female

1.20 Age of HH head (in years): ______________

1.21 Marital status of HH head: _______ [**1**] Single [**2**] Married [**3**] Divorced [**4**] Separated [**5**] Widowed [**6**]. Other_____________________

1.21 Educational level completed of HH head: _____ (1= Primary; 2= Junior High; 3= Senior High; 4= University; 5=Masters; 6=PhD)

1.22 Educational level of spouse: _____ (1= Primary; 2= Junior High; 3= Senior High; 4= University; 5=Masters; 6=PhD)

2. HOUSEHOLD DEMOGRAPHICS

2.1. Household size (total number of household members) #_____

Total number of members aged	Male	Female
Below 5 years	2.1.a)	2.1.b)
5 to 15 years	2.1.c)	2.1.d)
16 to 65 years	2.1.e)	2.1.f)
Above 65 years	2.1.g)	2.1.h)

2.2. What are the various sources of income or livelihood of your household?***(Circle All That Apply)***
1. Crop farming; **2.** Livestock farming **3.** Fish farming;; **4.** *Ganyu*/daily labor/piece work earner in agricultural sector; **5**. Wage earner in non-agriculture large business (factory operation); **6**. Petty business (kiosk, trade in farm produce); **7.** Artisan (e.g. welder, blacksmith, craftsman. Carpenter); **8.** Salaried employment/job; **9.** Remittances; **10.** Other (specify) ____.

2.2.a Do you have a home garden or cultivate your own fruit or vegetables? Yes (**1**) or No (**0**)
2.2.b If Yes, which fruit and/or vegetable crops do you grown and eat? _____________________________,

______________________________, ______________________________, ______________________________,

______________________________, ______________________________, ______________________________,

______________________________, ______________________________, ______________________________,

2.3. From **Q.2.2 above**, which are your three <u>major</u> sources of income for your household ?
(***1=most important)***)

Income source 1 Income source 2 Income source 3

[ ] [ ] [ ]

2.4. How do you use your income to achieve your household objectives?

Tick/check all mentioned objectives and rank ONLY 5 most important (1=most important, 5=least important)

**Objective**	**Rank**	**Achievement of the objective now:**<br>**1**=Easily achievable<br>**2**=No change<br>**3**=Increasingly difficult
2.4.a) Food		
2.4.b) Housing		
2.4.c) Transport		
2.4.d) School fees		
2.4.e) Medical care		
2.4.f) Purchase of fertilizers		
2.4.g) Purchase seed		
2.4.h) Purchase pesticides		
2.4.i) Hiring labour		
2.4.j) Others (specify)		

2.5.Are you able to save any money for unexpected expenses? **(*Circle one)*** **1**. Yes **0**. No

3. HOUSEHOLD LIVING CONDITIONS

3.1. Is there any expecting mother in this household? ***(Circle one)*1**. Yes; **0**. No

3.2. How many rooms are in your home, not including bathrooms, toilets, and the kitchen?

Number of rooms: _________

3.3. What kind of toilet facility do most members of your household usually use? ***(Circle One)***

1. Flush toilet; **2.** Ventilated improved latrine; **3.** Traditional latrine with roof; **4.**Traditional latrine without roof; **5.**No facility/bush/field; **6.** Other (specify) __________________

3.4. If answered "**No facility/bush/field**" to **Question 3.3**, does your household have:

3.4.a. Drying rack for plates & other household utensils? ***(Circle one)*** **1**. Yes; **0**. No

3.4.b. Pit for dumping wastes/rubbish Pit for dumping wastes/rubbish? ***(Circle one)*** **1**. Yes; **0**. No

3.4.c. Line for drying clothes? ***(Circle one)*** **1**. Yes; **0**. No

3.4.d Bathroom inside house? ***(Circle one)*** **1**. Yes; **0**. No

3.4.e Bathing area outside house ? ***(Circle one)*** **1**. Yes; **0**. No

3.5. Do you share your toilet facility with other households? ***(Circle one)*** **1**. Yes; **0**. No

3.6. If "**Yes**" to **Question 3.6**, how many households share this facility? ______

3.7. Do you have a hand-washing facility outside the toilet? ***Enumerator notes**: ASK & OBSERVE THE FACILITY.* ***(Circle one)*** **1.** Seen the facility filled with water; **2.** Seen the facility but no water; **3.** Not seen; **4.** No facility

3.8. ***Enumerator notes**: Ask to see the place most used for hand washing and observe if the following items are present **(Circle all that are present):*** **1.** Water/Tap; **2.** Tip-tap; **3.** Soap/ash/other cleansing agent; **4.** Basin (to wash hands inside); **5.** Basin for communal washing; **6.**Someone pouring water on others hand; **7.**Stone;

8.Other (SPECIFY____)

3.9. Do you have electricity working in your dwelling? ***(Circle one)*** **1**. Yes; **0**. No

4 HOUSEHOLD GENERAL FOOD CONSUMPTION FREQUENCIES

4.1. What is the frequency of consumption of the following foods by the members of the household?

**Food Item**	**Frequency of consumption per week**							
	Freq.*	Source	Adequate **1=Yes 0=No**	If Adequate is 0, what is frequency of shortage**?	Months when shortage occurs **CODE 1-12 for Jan to Dec**	Are you able to store this food in a separate storage area? **1= Yes 0= No**	If able to store food item: **- CODE FORM (dry=1, fresh=2, ground=3 other = 0)**	- for how long? **(indicate days, weeks of months with d, w, or m)**
	**1**	**2**	**3**	**4**	**5**	**6**	**7**	**8**
4.1.a) Green or dried maize/Nshima/ grits “samp”/ processed maize								
4.1.b) Maize/ millet/ cassava/ sorghum								
AIVs INDIGENOUS VEGETABLES								
c) Amaranth								
d) Nightshade								
e) Spider plant								
f) Cowpea								
g) Jute mallow								
h) Kale								
i) Sweet Potato Leaves								
j) Sweet Potato tubers								
k) Orange Sweet Potato leaves								
l) Orange Sweet Potato tubers								
m) Okra								
n) Ethiopian Mustard								
o) African Eggplant								

***Frequency: 0= Never; 1**=Rarely (once a month); **2**=Every day (6-7 times a week); **3**=Sometimes (1-2 times a week)

****Frequency of shortage: 1**=Every year; **2**=Once in 2 years; **3**=Once in 3 years;**4**=Once in 4 years; **5**=Once in 5 years

**Food Item**	**Frequency of consumption per week**							
	Freq.*	Source	Adequate **1=Yes 0=No**	If Adequate is 0, what is frequency of shortage**?	Months when shortage occurs **CODE 1-12 for Jan to Dec**	Are you able to store this food in a separate storage area? **1= Yes 0= No**	If able to store food item: **- CODE FORM (dry=1, fresh=2, ground=3 other = 0)**	- for how long? **(indicate days, weeks of months with d, w, or m)**
	**1**	**2**	**3**	**4**	**5**	**6**	**7**	**8**
p) Cassava leaves								
q) Cassava tubers								
r) Pumpkin leaves								
s) Lumanda (hibiscus sabdariffa)								
t) Mundambi (hibiscus roselle)								
u) Moringa								
v) Chikanda								
w) Blackjack plant								
x)								
y)								
Fruits (e.g mango, banana, oranges)								
aa)								
bb)								
cc)								
dd)								
ee) Legumes (e.g beans, peas)								
ff) Exotic vegetables (e.g. cabbage, lettuce, carrots etc)								
gg) Meat and meat products (e.g biltong, offals etc)								
hh) Milk								

***Frequency: 0=** Never; **1**=Rarely (once a month); **2**=Every day (6-7 times a week); **3**=Sometimes (1-2 times a week)

****Frequency of shortage: 1**=Every year; **2**=Once in 2 years; **3**=Once in 3 years;**4**=Once in 4 years; **5**=Once in 5 years

5 CONSUMPTION AND UTILIZATION OF AIVs

5.1. What is the main source of AIV for your household?(***Circle***)**1**. Own farm; **2**. Market; **3**. Gift; **4.**Other (specify)___________

5.2. If some AIVs are bought, what is the main place of buying? (***Circle***) **1.** Farm; **2.** Village market; **3.**Town/city market; **4.**Other (specify)___

5.3. Below, please state quantities of AIVs bought and consumed at home by the household in the past six months?

***0**=Never; **1**=Rarely (once a month or less); **2**=two to three times a month; **3**=Once/week; **4=**Twice/week; **5=**Three times a week; **6**=Four to Six times a week; **7=**Everyday (7 times a week)

**AIVs**		**Quantities of AIVs from various sources**			**Utilization**				
	Quantity harvested from wild, grown on own property (kg)	Quantities purchased (kg)	Price/kg If purchased	Purchasing frequency **(see codes above or below)**	Quantity of AIV per Meal (kg)	Key ingredients added to AIVs	How eaten? **1=Main dish 2=Side dish 3= Major ingredient in side dish 4=Condiments to staple food**	Frequency household eats AIV **(see codes above or below*)**	Quantity of AIV given as gift
	**1**	**2**	**3**	**4**	**5**	**6**	**7**	**8**	**9**
5.3.a) Amaranth									
5.3.b) Nightshade									
5.3.c) Spider plant									
5.3.d) Cowpea									
5.3.e) Jute mallow									
5.3.f) Kale									

5.3.g) Sweet Potato Leaves									
5.3.h)									
5.3.i)									
5.3.j)									

***0**=Never; **1**=Rarely (once a month or less); **2**=two to three times a month; **3**=Once/week; **4=**Twice/week; **5=**Three times a week; **6**=Four to Six times a week; **7=**Everyday (7 times a week)

6. HOUSEHOLD HUNGER SCALE			
**#**	**QUESTION AND FILTER**	**RESPONSE CODES**	**RESP**
6.1	In the past 4 weeks (30 days) was there ever no food to eat of any kind in your house because of lack of resources to get food?	Yes (1) _________<br>No (0) _________	
6.2	How often did this happen in the past 4 weeks (30 days)?	Rarely (1-2 times) [0]<br>Sometimes (3-10 times) [1]<br>Often (10+ times) [2]	
6.3	In the past 4 weeks (30 days) did you or any household member go to sleep at night hungry because there was not enough food?	Yes (1) _________<br>No (0) _________	
6.4	How often did this happen in the past 4 weeks (30 days)?	Rarely (1-2 times) [0]<br>Sometimes (3-10 times) [1]<br>Often (10+ times) [2]	
6.5	In the past 4 weeks (30 days), did you or any household member go a whole day and a night without eating anything at all because there was not enough food?	Yes (1) _________<br>No (0) _________	
6.6	How often did this happen in the past 4 weeks (30 days)?	Rarely (1-2 times) [0]<br>Sometimes (3-10 times) [1]<br>Often (10+ times) [2]	

**7. Consumer Attitude and Preferences**			
**#**	**QUESTION AND FILTER**	**RESPONSES**	
7.1	Which foods (of AIVs listed in Question 5.3) do you prefer to eat? (List all) (amaranth, nightshade, spiderplant, cowpea, jutemallow, kale, sweet potato leaves, orange sweet potato leaves, okra, Ethiopian mustard, African eggplant, other)	________________________________<br>________________________________<br>________________________________<br>________________________________<br>________________________________<br>________________________________<br>________________________________	
7.2	Why do you prefer the foods listed above? (Circle all that apply)	1. Affordable<br>2. Readily available/ easy to access<br>3. Have an abundance of this food<br>4. Taste<br>5. Easy to prepare/cook with<br>6. Health reasons<br>7. Other (specify)<br>_______________________________	
7.3	Would you be willing to consume AIVs on a daily basis if they were prepared in a better tasting meal? (Circle one)	Yes (1) __________ No (0) __________	
7.4	If you knew AIVs were healthier foods, would you be more willing to buy these food items? (Circle One)	Yes (1) __________ No (0) __________	

#	QUESTION AND FILTER		RESPONSE CODES
**8.WOMEN'S DIETARY DIVERSITY**<br>ASK THIS SECTION TO FEMALE PARTICIPANT OF REPRODUCTIVE AGE (18-59)			
	**RESPONDENT'S NAME AND LINE NUMBER**		**Pick from the HH roster**
8.1	How many meals and snacks did your household members eat yesterday during the day or at night?		NO OF MEALS/SNACKS ___<br>IF MORE THAN 7, RECORD '7'<br>IF NONE, RECORD '0'
8.2	Now I would like to ask you about the types of foods that you or anyone else in your household eat on a regular basis, day to day, week to week yesterday during the day or at night.<br><br>READ OUT EACH ITEM TO THE RESPONDENT AND RECORD		
	**a.** Any ugali, bread, rice, noodles, biscuits, or any other local foods made from millet, sorghum, maize, rice, wheat?		Yes (1) __________<br>No (0) __________<br>DK (3) __________
	**b.** Pumpkins, carrots, squash, orange/yellow fleshed sweet potatoes, or any other similar local foods		Yes (1) __________<br>No (0) __________<br>DK (3) __________
	**c.** White-fleshed sweet potatoes, potatoes, yams, manioc, cassava or any other foods made from roots or tubers?		Yes (1) __________<br>No (0) __________<br>DK (3) __________
	**d.** Dark green leafy vegetables		Yes (1) ___<br>No (0) ____<br>DK (3) ___
	e. Amaranth		Yes (1) ___<br>No (0) ____<br>DK (3) ___
	f. Nightshade		Yes (1) ___<br>No (0) ____<br>DK (3) ___
	g. Spider plant		Yes (1) ___<br>No (0) ____<br>DK (3) ___

	h. Cowpea	Yes (1) ___<br>No (0) ____<br>DK (3) ___
	i. Jute mallow	Yes (1) ___<br>No (0) ____<br>DK (3) ___
	j. Kale	Yes (1) ___<br>No (0) ____<br>DK (3) ___
	k. Sweet Potato Leaves	Yes (1) ___<br>No (0) ____<br>DK (3) ___
	l. Orange Sweet Potato	Yes (1) ___<br>No (0) ____<br>DK (3) ___
	m. Okra	Yes (1) ___<br>No (0) ____<br>DK (3) ___
	n. Ethiopian Mustard	Yes (1) ___<br>No (0) ____<br>DK (3) ___
	o. African Eggplant	Yes (1) ___<br>No (0) ____<br>DK (3) ___
	**p.** Ripe mangoes, ripe papayas or any other fruits that are rich in vitamin A?	Yes (1) ___<br>No (0) ____<br>DK (3) ___
	**q.** Any other fruits or vegetables?	Yes (1) ___<br>No (0) ____<br>DK (3) ___
	r. Liver, kidney, heart or other organ meats	Yes (1) ___<br>No (0) ____<br>DK (3) ___
	s. Any beef, pork, lamb, goat, rabbit, wild game, chicken, duck, mice or other birds?	Yes (1) ___<br>No (0) ____<br>DK (3) ___
	t. Any eggs?	Yes (1) ___<br>No (0) ____<br>DK (3) ___

	u. Any fresh or dried fish, shellfish or sea foods?	Yes (1) ___<br>No (0) ____<br>DK (3) ___
	**v.** Any foods made from beans, peas, lentils, nuts or seeds? (local names)	Yes (1) ___<br>No (0) ____<br>DK (3) ___
	**w.** Any cheese, yogurt, milk or other milk products?	Yes (1) ___<br>No (0) ____<br>DK (3) ___
	**x.** Any foods made with oil, fat, or butter?	Yes (1) ___<br>No (0) ____<br>DK (3) ___
	y. Any sugar or sugary foods such as candies, pastries, chocolates?	Yes (1) ___<br>No (0) ____<br>DK (3) ___
	z. Any other foods, such as condiments, coffee, tea?	Yes (1) ___<br>No (0) ____<br>DK (3) ___
	aa. Grubs, snails, or insects	Yes (1) ___<br>No (0) ____<br>DK (3) ___
	bb. Food made with red palm oil, red palm nut, or red palm nut pulp sauce	Yes (1) ___<br>No (0) ____<br>DK (3) ___
	zz. Others (list) (ENSURE THEY DO NOT FALL WITHIN LISTS PROVIDED ABOVE)	Yes (1) ___<br>No (0) ____<br>DK (3) ___

**9. ROLE IN HOUSEHOLD DECISION-MAKING AROUND PRODUCTION AND INCOME GENERATION** RESPONDENT ID						
"Now I'd like to ask you some questions about your participation in certain types of work activities and on making decisions on various aspects of household life"		Did you yourself participate in [ACTIVITY] in the past 12 months (that is, during the last [one/two] cropping seasons), from [PRESENT MONTH] last year to [PRESENT MONTH] this year?	When decisions are made regarding [ACTIVITY], who is it that normally makes the decision?<br><br>CIRLCE ALL APPLICABLE<br><br>IF THE RESPONSE IS SELF ONLY SKIP TO QUESTION 9.05	How much input did you have in making decisions about [ACTIVITY]?<br>USE DECISION CODES FOR 9.03/9.05;<br>IF NO DECISION MADE, ENTER 98 AND MOVE TO THE NEXT ACTIVITY	To what extent do you feel you can make your own personal decisions regarding [ACTIVITY] if you want(ed) to?<br><br>CIRCLE ONE	How much input did you have in decisions on the use of income generated from [ACTIVITY]?<br><br>USE CODES FOR 9.03/9.05
**ACTIVITY CODE**	**ACTIVITY DESCRIPTION**	**9.01**	**9.02**	**9.03**	**9.04**	**9.05**
**A**	Food crop farming: these are crops that are grown primarily for household food consumption	YES ....1<br>NO ....2 → *ACTIVITY B*	SELF .... 1<br>SPOUSE .... 2<br>OTHER HH MEMBER .... 3<br>OTHER NON-HH MEMBER ...... 4<br>NOT APPLICABLE .... 98 →<br>*NEXT ACTIVITY*		NOT AT ALL .... 1<br>SMALL EXTENT .... 2<br>MEDIUM EXTENT .... 3<br>TO A HIGH EXTENT 4	
**B**	Cash crop farming: These are crops that are grown primarily for sale in the market	YES ....1<br>NO ....2 → *ACTIVITY C*	SELF .... 1<br>SPOUSE .... 2<br>OTHER HH MEMBER .... 3<br>OTHER NON-HH MEMBER ...... 4<br>NOT APPLICABLE .... 98 →<br>*NEXT ACTIVITY*		NOT AT ALL .... 1<br>SMALL EXTENT .... 2<br>MEDIUM EXTENT .... 3<br>TO A HIGH EXTENT 4	
**C**	Livestock raising	YES ....1<br>NO ....2 → *ACTIVITY D*	SELF .... 1<br>SPOUSE .... 2<br>OTHER HH MEMBER .... 3<br>OTHER NON-HH MEMBER ...... 4<br>NOT APPLICABLE .... 98 →<br>*NEXT ACTIVITY*		NOT AT ALL .... 1<br>SMALL EXTENT .... 2<br>MEDIUM EXTENT .... 3<br>TO A HIGH EXTENT 4	

**D**	Non-farm economic activities: This would include things like running a small business, self-employment, buyand-sell	YES ....1<br>NO ....2 → *ACTIVITY E*	SELF .... 1<br>SPOUSE .... 2<br>OTHER HH MEMBER .... 3<br>OTHER NON-HH MEMBER ...... 4<br>NOT APPLICABLE .... 98 →<br>*NEXT ACTIVITY*		NOT AT ALL .... 1<br>SMALL EXTENT .... 2<br>MEDIUM EXTENT .... 3<br>TO A HIGH EXTENT 4	

9.03/9.05 DECISION CODES:
NO INPUT OR INPUT IN FEW DECISIONS 01
INPUT INTO SOME DECISIONS 02
INPUT INTO MOST OR ALL DECISIONS 03
NO DECISION MADE 98

ACTIVITY CODE	ACTIVITY DESCRIPTION	9.01	9.02	9.03	9.04	9.05
**E**	Wage and salary employment: This could be work that is paid for in cash or in-kind, including both agriculture and other wage work	YES .... 1<br>NO .... 2 → *ACTIVITY F*	SELF .... 1<br>SPOUSE .... 2<br>OTHER HH MEMBER .... 3<br>OTHER NON-HH MEMBER ...... 4<br>NOT APPLICABLE .... 98 →<br>*NEXT ACTIVITY*		NOT AT ALL .... 1<br>SMALL EXTENT .... 2<br>MEDIUM EXTENT .... 3<br>TO A HIGH EXTENT 4	
**F**	Fishing or fishpond culture	YES .... 1<br>NO .... 2 → *ACTIVITY G*	SELF .... 1<br>SPOUSE .... 2<br>OTHER HH MEMBER .... 3<br>OTHER NON-HH MEMBER ...... 4<br>NOT APPLICABLE .... 98 →<br>*NEXT ACTIVITY*		NOT AT ALL .... 1<br>SMALL EXTENT .... 2<br>MEDIUM EXTENT .... 3<br>TO A HIGH EXTENT 4	
**G**	Major household expenditures (such as bicycles, land, boda boda)		SELF .... 1<br>SPOUSE .... 2<br>OTHER HH MEMBER .... 3<br>OTHER NON-HH MEMBER ...... 4<br>NOT APPLICABLE .... 98 →<br>*NEXT ACTIVITY*		NOT AT ALL .... 1<br>SMALL EXTENT .... 2<br>MEDIUM EXTENT .... 3<br>TO A HIGH EXTENT 4	
**H**	Minor household expenditures (such as food for daily consumptions or other household needs)		SELF .... 1<br>SPOUSE .... 2<br>OTHER HH MEMBER .... 3<br>OTHER NON-HH MEMBER ...... 4<br>NOT APPLICABLE .... 98 →<br>*MODULE G3 (A)*		NOT AT ALL .... 1<br>SMALL EXTENT .... 2<br>MEDIUM EXTENT .... 3<br>TO A HIGH EXTENT 4	

9.03/9.05 DECISION CODES:
NO INPUT OR INPUT IN FEW DECISIONS 01
INPUT INTO SOME DECISIONS 02
INPUT INTO MOST OR ALL DECISIONS 03
NO DECISION MADE 98

10. Qualitative Dietary Change Questions

Directions: Transcribe the respondent's answers as accurately as possible in the space provided - do not summarize. If more space is required, use the back of the survey and indicate which question the answer corresponds to.

	**Questions**	**Answer**
10	Do you think your diet has changed in the past 6 months?<br>**If YES - Skip to Question 1.a**<br>**If NO - Skip to Question 1.f**	*Circle:* YES or NO
10.a	**If YES to Q1:** How has it changed?	*Write answer in space below:*
10.b	**If YES to Q1:** Why has it changed?	*Write answer in space below:*
10.c	**If YES to Q1:** What influenced the changes to your diet the most?	*Write answer in space below:*
10.d	**If YES to Q1:** Are there ways you would like your diet to change?	*Circle:* YES or NO
10.e	**If YES to Q1:** What prevents you from making these changes?	*Write answer in space below:*
10.f	**If NO to Q1:** Do you want it to change?	*Circle:* YES or NO
10.g	**If NO to Q1:** What prevents you from making these changes?	*Write answer in space below:*
